# 913 Cost-effectiveness of Native Collagen-elastin Dermal Regeneration Template in Chronic and Acute Wounds

**DOI:** 10.1093/jbcr/iraf019.444

**Published:** 2025-04-01

**Authors:** Markus Oehlbauer

**Affiliations:** Burn Center BG Trauma Center Murnau

## Abstract

**Introduction:**

An acellular single-layer dermal substitute composed of native bovine collagen (types I, III, and V) and elastin hydrolysate can be used in single-step and two-step reconstruction surgeries in several skin defects. To our knowledge, there is a lack of cost-effectiveness analysis of native collagen-elastin dermal regeneration template compared to other dermal templates in wound treatment. The objective of this study is to assess the cost-effectiveness of native collagen-elastin dermal regeneration template in the treatment of both acute and chronic wounds from the perspective of the healthcare systems, considering cost factors such as material cost, hospitalization time and complications.

**Methods:**

The analyses were performed comparing native collagen-elastin dermal regeneration template with split-thickness skin graft (STSG) alone and other dermal templates. Diabetic foot ulcer (DFU) and burns were the indications selected to represent chronic and acute wounds, respectively. Separate mathematical models were developed for each indication: a Markov model for DFU and a decision tree model for burns. The Markov model for DFU was comprised of six health states: DFU (initial state); infected DFU; healed; amputation; infected post-amputation and death. The burns decision tree model had two health states: healed wound and regraft. Transition probabilities were obtained through literature search and from clinical studies of each of the comparators.

**Results:**

Native collagen-elastin dermal regeneration template was cost-effective in all comparisons, leading to savings of GBP 39,879 when compared to other dermal templates in acute wounds with marginal quality-adjusted life-year (QALY) gains). In chronic wounds native collagen-elastin dermal regeneration template led to savings and QALY gains against all comparators with incremental QALYs: 0.03, 0.14, 0.15; and cost savings: GBP 2,535; GBP 72,726; and GBP 4,465, compared to STSG, other dermal templates and outpatient templates, respectively.

**Conclusions:**

Highlighting the importance of a holistic economic approach considering cost factors as material costs, hospitalization time and complications native collagen-elastin dermal regeneration template has proven to be more effective and less costly (i.e. dominant) than all comparators. As the clinical data is obtained from heterogeneous populations and varying wound characteristics, the obtained can be seen as an indicator.

**Applicability of Research to Practice:**

Assessment of cost-effectiveness for different dermal regeneration templates considering cost factors such as material cost, hospitalization time and complications has proven major impact for the healthcare systems in the treatment of both acute and chronic wounds

**Funding for the Study:**

N/A

